# Subclinical systolic and diastolic myocardial dysfunction in polyphasic polymyositis/dermatomyositis: a 2-year longitudinal study

**DOI:** 10.1186/s13075-022-02906-7

**Published:** 2022-09-10

**Authors:** Andrea Péter, Ágnes Balogh, Zoltán Csanádi, Katalin Dankó, Zoltan Griger

**Affiliations:** 1 Division of Cardiology, Department of Cardiology and Cardiac Surgery, Faculty of Medicine, University of Debrecen, Debrecen, Hungary; 2 Division of Clinical Immunology, Institute of Internal Medicine, Faculty of Medicine, University of Debrecen, Debrecen, Hungary

**Keywords:** Polymyositis, Dermatomyositis, Echocardiography, Tissue Doppler imaging, Cardiac involvement

## Abstract

**Background:**

Cardiac involvement in patients with idiopathic inflammatory myopathies (IIM) is associated with increased morbidity and mortality risk; however, little is known about the progression of cardiac dysfunction and long-term data are scarce. In the present work, we intended to prospectively study echocardiographic parameters in patients with IIM for 2 years.

**Methods:**

Twenty-eight IIM patients (41.9±1.6 years) without cardiovascular symptoms were enrolled. Patients with monophasic/polyphasic disease patterns were studied separately and compared to age-matched healthy individuals. Conventional echocardiographic and tissue Doppler imaging (TDI) parameters of systolic [LV: ejection fraction (EF), mitral annulus systolic movement (MAPSE), lateral s′) and diastolic left (mitral inflow velocities, lateral anulus velocities: e′, a′, E/e′) and right ventricular function (fractional area change: FAC, tricuspid annulus plane systolic excursion: TAPSE) were measured at the time of the diagnosis and 2 years later.

**Results:**

Subclinical LV systolic dysfunction is characterized by reduced lateral s′ (10.4 vs. 6.4 cm/s, *p*<0.05), EF (62.6±0.6%, vs. 51.7±0.7%) and MAPSE (18.5±0.6 vs. 14.5±0.6 mm) could be observed in IIM patients with polyphasic disease course 2 years after diagnosis compared to controls. Furthermore, diastolic LV function showed a marked deterioration to grade I diastolic dysfunction at 2 years in the polyphasic group (lateral e′: 12.9 ±0.6, vs. 7.4±0.3 cm/s; lateral a′: 10.7±0.3, vs. 17.3±0.8 cm/s; *p*<0.05) supported by larger left atrium (32.1±0.6 vs. 37.8±0.6 mm; *p*<0.05]. TDI measurements confirmed subclinical RV systolic dysfunction in polyphasic patients 2 years after diagnosis (FAC: 45.6±1.8%, vs. 32.7±1.4%; TAPSE: 22.7±0.5, vs. 18.1±0.3 mm; *p*<0.05). Similar, but not significant tendencies could be detected in patients with monophasic disease patterns. Polyphasic patients showed significantly (*p*<0.05) worse results compared to monophasic patients regarding EF (51.7±0.7% vs. 58.1±0.6%), lateral s′ (6.4±0.4 cm/sec vs. 8.6±0.4 cm/s,), left atrium (37.8±0.6 mm vs. 33.3±0.8 mm), FAC (32.7±1.4% vs. 41.0±1.6%) and TAPSE (18.1±0.3 mm vs. 21.3±0.7 mm).

**Conclusions:**

Significant subclinical cardiac dysfunction could be detected in IIM patients with polyphasic disease course 2 years after diagnosis, which identifies them as a high-risk population. TDI is a useful method to detect echocardiographic abnormalities in IIM complementing conventional echocardiography and can recognize the high cardiac risk.

## Background

Idiopathic inflammatory myopathies (IIM), such as polymyositis and dermatomyositis (PM/DM), are chronic immune-mediated diseases, which are associated with inflammatory cell infiltration, destruction and fibrosis of the muscle fibres resulting in progressive weakness of the proximal muscles. Since 2017, the EULAR/ACR Classification criteria are most widely used for the diagnosis of PM/DM [1]. Extramuscular organ involvement is also frequently present; moreover, malignancies and pulmonary and cardiac complications account for the bulk of the mortality in these patients [2]. Numerous diagnostic approaches (autopsy, myocardial biopsy, histology, echocardiography, cardiac MRI - CMR) have already confirmed the myocardial manifestation over the skeletal muscles in the underlying inflammatory process. The degree of the myocardial involvement may be different according to the disease subtypes or the disease phases; however, many studies confirmed it as the predominant prognostic factor of the survival [3–6]. The accurate incidence of cardiac abnormalities of patients with IIM is unknown, thus the results vary between 9 and 72%. Zhang’s meta-analysis reported that heart disease was the cause of death in 46.3% of the patients [6].

Echocardiography, e.g. tissue Doppler imaging (TDI) and speckle tracking, are suitable for the recognition of both the early subclinical and the subsequent more severe, possibly life-threatening cardiac manifestations like progressive myocarditis [7], cardiomyopathy [8], rupture of the chordae tendineae [9], right heart failure [10] or restrictive cardiomyopathy [11]. The early alterations revealed at the time of diagnosis by conventional echocardiography, TDI or CMR include subclinical left and right ventricular dysfunction with myocardial oedema and acute myocarditis as the underlying mechanism [12, 13]. Specific steroid therapy can lead to the improvement or the normalization of the systolic dysfunction; however, asymptomatic diastolic dysfunction may occur from the third month after the diagnosis [13]. The presence of the diastolic dysfunction has also been reported in middle-aged patients without cardiovascular (CV) risk factors during the first year of the disease [14, 15]. The recently published guideline on management of patients with idiopathic inflammatory myopathy clearly postulate that patients should undergo a regular cardiovascular risk assessment and screening for cardiac involvement [16]. However, long term follow-up studies lack in the IIM population and the exact frequencies and the order of imaging modalities (i.e. echocardiography, TDI, speckle tracking, CMR), which have different advantages and disadvantages need further clarification.

Therefore, we planned a prospective 2-year follow-up echocardiographic study using TDI in patients specifically treated for IIM without CV risk factors. The aim of this study was to recognize the changes in the systolic and diastolic function and to find possible differences in the different subtypes of the disease course (monophasic, polyphasic), to identify high-risk populations.

## Methods

### Study population

Thrity hospitalized PM/DM patients (23/7) were consecutively enrolled with mean age of 42.3±1.6 years (27/3 female/male) without cardiovascular symptoms. Traditional echocardiography and TDI were performed to measure systolic and diastolic echocardiographic variables at the time of diagnosis, 3 months and 2 years later. All patients met the Bohan and Peter [17] and/or 2017 EULAR/ACR classification criteria for definitive PM or DM [1]. Patients presented with malignancy, overlap syndromes, previously diagnosed congenital heart disease, rheumatic fever, hypertension, coronary artery disease, atrial fibrillation, mitral regurgitation exceeding moderate severity, diabetes mellitus, cardiomyopathy, severe renal disease (serum creatinine level≥130 μmol/l) or anaemia (hemoglobine≤13.5 g/dl in male, ≤12.0 g/dl in female) were excluded. Characteristics of the first 3 months of treatment were published earlier [13]. During the follow-up period, 2 female patients were excluded from the study, one had new ECG abnormalities (RV strain signs) with PM/SSc overlap syndrome and pulmonary hypertension; moreover, breast cancer occurred in the other patient baseline results represent the 3 months data of the 28 remaining patients. On the basis of the disease progress, the patients were classified into two groups: monophasic (*n*=16) and polyphasic (*n*=12) groups. Patients with monophasic disease course did not experience disease flare and could adhere to steroid tapering, whereas patients with polyphasic disease course experienced disease relapse during the follow-up period. Echocardiographic findings were compared in the different groups and to an age- and sex-matched healthy control group (*n*=26).

High-resolution computed tomography of the lungs was performed to investigate radiographic abnormalities [pulmonary infections, fibrosis, tuberculosis, and interstitial lung disease (ILD)]. Autoimmune panel (anti-dsDNA, anti-SRP, anti-Scl-70, and myositis profile 3 Blot Strip: Ro52, OJ, EJ, Pl-12, Pl-7, SRP, anti-Jo1, PM-Scl75, PM-Scl100, Ku, and Mi-2B) was performed at the diagnosis and was re-evaluated during the follow-up by membrane-fixed line blots (Euroline Myositis Antigen Profile4, EuroImmun, Lübeck, Germany) according to manufacturer’s instructions.

### Echocardiography

Traditional echocardiographic measurements and TDI were performed at the time of the diagnosis and at the end of the follow-up period. Transthoracic echocardiography was performed using an ultrasound equipment (Accuson Sequoia) with a 1–5-MHz transducer. All measurements were performed in adherence with the guidelines and standards of the European Society of Echocardiography [18] by a single observer blinded to patient/control status. All measurements were taken on 3 consecutive beats, and the mean values were used. The study protocol was approved by the Ethics Committee of the University of Debrecen (28192/2011-EKU) and written informed consent was obtained from each participant. The study was conducted according to the Declaration of Helsinki (2000).

### General parameters

Left atrial (LA) diameter, left ventricular (LV) end-diastolic (LVEDD) and LV end-systolic diameters (LVESD), thickness of the interventricular septum (IVS), and right atrial (RA) and right ventricular (RV) diameters were measured using 2D and M-mode echocardiography based on the criteria of the European Society of Echocardiography [18].

### Left ventricular systolic function

LV systolic function was characterized both by conventional echocardiographic and TDI parameters. Left ventricular ejection fraction (EF) was assessed according to the biplane Simpson’s method [18]. This method calculates LV volumes by tracing the endocardial borders in apical 4- and 2-chamber views in end-diastole and end-systole. Mitral annular plane systolic excursion (MAPSE) measured in M-mode characterizes the longitudinal function of the left ventricle, and it is an accurate predictor of EF. MAPSE was calculated by placing M-mode cursor through the mitral annulus in a standard apical 4-chamber window and measuring the difference between end-diastolic and end-systolic amount of longitudinal motion of the annulus. The peak myocardial systolic velocity (s) of the lateral site of the mitral annulus (lateral s′) obtained by the TDI method was also used for the evaluation of the LV systolic function.

### LV diastolic function

Mitral inflow velocities were evaluated by pulsed wave Doppler imaging with the sample volume placed at the tip of the mitral leaflets that can be seen on the apical 4-chamber view. Deceleration time of the E wave (DT), peak early (E) and peak late diastolic transmitral flow velocities (A) were measured to calculate the ratio of peak E to peak A velocities (E/A) for the characterization of the diastolic function. The early myocardial diastolic velocity (e′) and the late myocardial diastolic velocity (a′) measured at the lateral site of the mitral annulus by the TDI method are also indices of left ventricular diastolic function, and E/e′ ratio can be applied for the estimation of left ventricular filling pressure.

### RV function

RV function was calculated according to the fractional area change (FAC: end-diastolic RV area – end-systolic RV area/end-systolic RV area). Tricuspid annular plane systolic excursion (TAPSE) was measured by M-mode to calculate the longitudinal function of the right ventricle. TAPSE was calculated by placing M-mode cursor through the tricuspid annulus in a standard apical 4-chamber window and measuring the difference between the end-diastolic and end-systolic amount of longitudinal motion of the annulus. TDI data were obtained from the tricuspid annulus measuring peak myocardial systolic velocity (tricuspid s′), early myocardial diastolic velocity (tricuspid e′) and late myocardial diastolic velocity (tricuspid a′). Systolic pulmonary artery pressure (sPAP) was calculated from the maximal tricuspid regurgitation velocity and the estimated RA pressure [18].

### Statistical analysis

Numerical data in this study are given as mean values±SEM. The values of continuous variables in different groups were compared with either the nonparametric Kruskal-Wallis test or analysis of variance (ANOVA) followed by the Bonferroni post hoc test, depending on the result of the normality test (Shapiro-Wilk, alpha=0.05). *P* values <0.05 were considered statistically significant. Statistical analyses were performed with GraphPad Prism 5.02 software (GraphPad Software, Inc., La Jolla, CA, USA).

## Results

### Clinical characteristics

Data of 28 IIM patients were evaluated, who completed the study. Table 1 shows the demographic, clinical, and serological characteristics of the patients. The mean age at diagnosis was 41.9 ± 1.6 years, the female/male ratio was 25/3. Autoimmune profile confirmed at the time of the diagnosis with myositis profile 3 Blot Strip (anti-Jo 1: 6/28, anti-PM/Scl-100: 1/28, anti-PM-scl-75: 3/28) was re-evaluated with line blot assay (Euroline Myositis Antigen Profile4) and we could detect 4 new autoantibody positivity (1 anti-Mi2, 1 anti-TIF1γ, 2 anti-NXP2). New therapy was released during the 24 months as follows: methotrexate in 5 cases, cyclophosphamide in 2 cases and rituximab in 2 cases; however, steroid was administered in 100% of the cases. The mean blood pressure (130/78 mmHg) was slightly higher at the end of the follow-up period, compared to baseline and control; hence, 7/28 patients had mild hypertension at the end of the study. New-onset diabetes could not be detected during the follow-up period.

**Table 1:** Demographic, clinical, and serological characteristics of the study population

	IIM baseline; *n*=28	IIM 2 years; *n*=28	Control; *n*=26
**Demography, organ involvements**			
Mean age at onset, years, Mean ± SEM	41.9 ± 1.6	NA	43.7 ± 0.7
Sex (female/male) *n*	25/3 (89/11%)	25/3 (89/11%)	23/3 (88/12%)
Diagnosis (PM/DM)	21/7 (77%/23%)	21/7 (77%/23%)	NA
Interstitial lung disease	35.7%	35.7%	NA
Raynaud sign	32.1%	32.1%	NA
Dysphagia	14.2%	14.2%	NA
Arthritis	71.4%	71.4%	NA
SBP, mmHg	120 ± 2	130 ±1	122 ±2
DBP, mmHg	76 ± 1	78±1	73±1
HR, beat/min	85 ± 1	79±2	76±3
**Antibodies**	Myositis profile 3 Blot Strip	Euroline Myositis Antigen Profile4	NA
Anti-Jo1	6	6	NA
Anti-PL7	0	0	NA
Anti-PL12	0	0	NA
Anti-EJ	0	0	NA
Anti-OJ	0	0	NA
Anti-SRP	0	0	NA
Anti-Mi2	0	1	NA
Anti-NXP2	0	2	NA
Anti-MDA5	0	0	NA
Anti-TIF1gamma	0	1	NA
Anti-Pm/scl-100	1	1	NA
Anti-Pm/scl-75	3	3	NA
Anti-Ku	0	0	NA
Anti-Ro52	6	6	NA
Anti-DNS	0	0	NA
**Immunsupressive therapy**			
Corticosteroid	28	28	0
Cyclosporin-A	4	4	0
Methotrexate	0	5	0
Cyclophosphamid	0	2	0
Rituximab	0	2	0
**Disease course**			
Monophasic (without relapse) *n*	NA	16	NA
Polyphasic (with relapse) *n*	NA	12	NA

*PM* polymyositis (including necrotizing myopathy), *DM* dermatomyositis, *SBP* systolic blood pressure, *DBP* diastolic blood pressure, *HR* heart rate, *NA* not applicable

### Echocardiographic findings

The results of the echocardiographic parameters during the follow-up are presented in Table 2. The right atrial (RA: 29.9±0.5 mm, 30.5±0.7 mm, 31.9±1.1 mm, 29.9±0.8 mm) and right ventricular dimensions (RV1: 25.6±0.3 mm, 26.7±0.8 mm, 26.8±1.3 mm, 27.8±1.4 mm; RV2: 26.0±0.7 mm, 25.2±0.7 mm, 28.1±2.4 mm, 27.2±1.1 mm; RV3: 55.6±1.1 mm, 57.3±1.8 mm, 56.5±2.4 mm, 50.3±2.8 mm; RVSA: 9.4±0.3 cm^2^, 11.0±0.8 cm^2^, 9.3±0.4 cm^2^, 9.3±0.5 cm^2^; RVDA: 17.1±1.1 cm^2^, 16.5±0.6 cm^2^, 16.6±0.7 cm^2^, 17.3±0.9 cm^2^; control, baseline, monophasic, polyphasic, respectively) were in the normal range and did not change compared either to the controls or to the baseline during the follow-up. LV diameters were also in the normal range. We could not detect any significant changes in LVESD (30.0±0.9 mm, 28.2±0.8 mm, 34.6±2.4 mm, 30.3±1.4 mm; control, baseline, monophasic, polyphasic, respectively), or in the LVEDD (49.3±1.0 mm, 47.1±1.1 mm 44.0±1.8 mm, 50.3±1.4 mm; control, baseline, monophasic, polyphasic, respectively).

**Table 2:** Echocardiographic variables of IIM patients during 2 years of follow-up.

Echocardiographic variable	Healthy control (*n*=26)	Baseline (*n*=28)	Monophasic (*n*=16)	Polyphasic (*n*=12)	*P* value					
		3 months	2 years	2 years	Control vs. Baseline	Control vs. Monophasic	Control vs. Polyphasic	Baseline vs. Monophasic	Baseline vs. Polyphasic	Monophasic vs. Polyphasic
LA (mm)	32.1±0.6	32.2±0.7	33.3±0.8	37.8±0.6	n.s.	n.s.	****	n.s.	****	**
LVESD (mm)	30.0±0.9	28.2±0.8	34.6±2.4	30.3±1.4	n.s.	n.s.	n.s.	n.s.	n.s.	n.s.
LVEDD (mm)	49.3±1.0	47.1±1.1	44.0±1.8	50.3±1.4	n.s.	n.s.	n.s.	n.s.	n.s.	n.s.
EF (%)	62.6±0.6	60.9±0.9	58.1±0.6	51.7±0.7	n.s.	***	***	n.s.	***	***
MAPSE (mm)	18.5±0.6	18.0±0.7	17.7±1.0	14.5±0.6	n.s.	n.s.	**	n.s.	*	n.s.
lateral s′ (cm/s)	10.4±0.3	8.6±0.4	8.6±0.4	6.4±0.4	**	**	****	n.s.	***	**
E/A	1.33±0.02	1.32±0.1	0.84±0.06	0.68±0.04	n.s.	****	****	****	****	n.s.
DT (msec)	144.7±3.2	158.3±5.7	182.8±15.4	190.8±7.6	n.s.	**	**	n.s.	n.s.	n.s.
E/e′	5.8±0.2	5.0±0.2	8.7±0.6	9.0±0.4	n.s.	****	****	****	****	n.s.
lateral e′ (cm/s)	12.9±0.2	12.3±0.6	8.7±0.9	7.4±0.3	n.s.	***	***	***	***	n.s.
lateral a′ (cm/s)	10.7±0.3	11.1±0.8	15.4±1.2	17.3±0.8	n.s.	***	****	**	****	n.s.
RA (mm)	29.9±0.5	30.5±0.7	31.9±1.1	29.9±0.8	n.s.	n.s.	n.s.	n.s.	n.s.	n.s.
RV1 (mm)	25.6±0.3	26.7±0.8	26.8±1.3	27.8±1.4	n.s.	n.s.	n.s.	n.s.	n.s.	n.s.
RV2 (mm)	26.0±0.7	25.2±0.7	28.1±2.4	27.2±1.1	n.s.	n.s.	n.s.	n.s.	n.s.	n.s.
RV3 (mm)	55.6±1.1	57.3±1.8	56.5±2.4	50.3±2.8	n.s.	n.s.	n.s.	n.s.	n.s.	n.s.
RVSA (cm^2^)	9.4±0.3	11.0±0.8	9.3±0.4	9.3±0.5	n.s.	n.s.	n.s.	n.s.	n.s.	n.s.
RVDA (cm^2^)	17.1±1.1	16.5±0.6	16.6±0.7	17.3±0.9	n.s.	n.s.	n.s.	n.s.	n.s.	n.s.
FAC (%)	45.6±1.8	37.0±1.5	41.0±1.6	32.7±1.4	***	n.s.	****	n.s.	*	*
TAPSE (mm)	22.7±0.5	22.3±0.7	21.3±0.7	18.1±0.3	n.s.	n.s.	***	n.s.	***	*
tricuspid s′ (cm/sec)	13.1±0.3	9.6±0.4	9.3±0.5	7.8±0.2	****	****	****	n.s.	*	n.s.
tricuspid e′ (cm/sec)	13.3±0.5	10.7±0.6	9.4±0.7	7.2±0.3	**	****	****	n.s.	**	n.s.
tricuspid a′ (cm/s)	11.5±0.4	14.6±0.9	14.3±0.9	15.1±0.7	**	n.s.	*	n.s.	n.s.	n.s.

*EF* Ejection fraction, *MAPSE* Mitral annulus plane systolic excursion,*lateral s* Peak systolic mitral annulus velocity, *E* Peak early diastolic velocity, *A* Peak late diastolic velocity, *DT* Deceleration time, *lateral e′* Early myocardial diastolic velocity, *lateral a′* Late myocardial diastolic velocity, *LA* Left atrial diameter, *FAC* Fractional area change, *tricuspid e′* Early tricuspid annular diastolic velocity, *tricuspid s* Peak systolic tricuspid annulus velocity, *tricuspid a′* Late tricuspid annular diastolic velocity, *TAPSE* Tricuspid annulus plane systolic excursion, *RA* Right atrial diameter, *LVEDD* Left ventricular end-diastolic diameter, *LVESD* Left ventricular end-systolic diameter, *RV* Right ventricular diameter measured at 3 different levels, *RVSA* Right ventricular systolic area, *RVDA* Right ventricular diastolic area; *p* value: ≥0.05: non-significant (n.s.); 0.01-0.05: *; 0.001–0.01: **; 0.0001–0.001: ***; <0.0001: ****

### LV systolic function

**Fig. 1:**
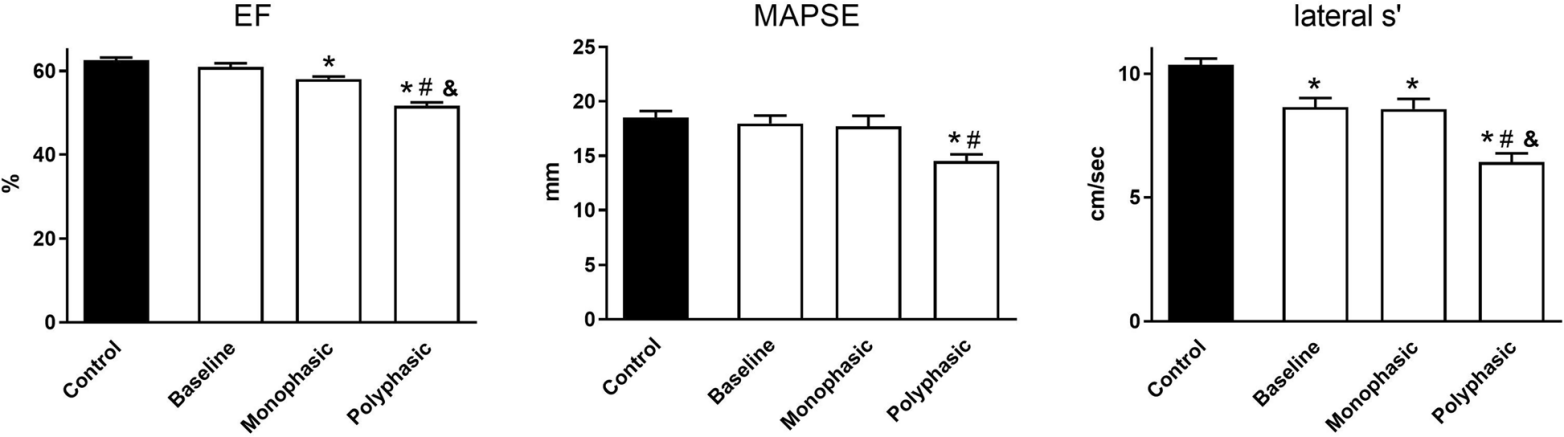
Two-year follow-up of the left ventricular systolic function. EF, ejection fraction; MAPSE, mitral annulus systolic motion; lateral s′, tissue Doppler imaging systolic wave velocity. EF measured by the Simpson’s method suggested normokinetic left ventricular systolic function at baseline and during the follow-up period in the monophasic group (significantly lower, but normal EF); however, it was lower in the polyphasic group compared to the other groups. In line, MAPSE was in the normal range at baseline and during the follow-up in the monophasic group; however. it was found to be significantly lower in the polypasic group compared to the control and the baseline group. Additionally, a lower lateral s′ wave velocity showed LV systolic dysfunction already at baseline and at the end of the follow-up period in both groups, as well. * *P*<0.05 vs. control, # *P*<0.05 vs. baseline, & *P*<0.05 vs. monophasic

Global systolic function of the LV was characterized by the traditionally used parameter, the EF measured by the Simpson’s method. We detected a significantly impaired LVEF in both subgroups at the end of the follow-up compared to the controls (Table 2; Fig. 1); however, it was more pronounced in the polyphasic group where it was significantly lower than the baseline or the monophasic group (62.6±0.6%, 60.9±0.9%, 58.1±0.6%, 51.7±0.7%; control, baseline, monophasic, polyphasic, respectively). The longitudinal left ventricular systolic motion (MAPSE) assessed by M-mode decreased significantly during the 2 years in patients with polyphasic disease patterns compared to the baseline and the control group (18.5±0.6 mm, 18.0±0.7 mm, 17.7±1.0 mm, 14.5±0.6 mm; control, baseline, monophasic, polyphasic, respectively). LV systolic function measured by the TDI method showed major changes: the mitral lateral systolic velocity (lateral s′) was significantly lower in both subgroups at 2 years than at the time of the diagnosis; moreover, the polyphasic group was found to have a remarkable decreased s′ velocity (10.4±0.3 cm/s, 8.6±0.4 cm/s, 8.6±0.4 cm/s, 6.4±0.4 cm/s; control, baseline, monophasic, polyphasic, respectively). The above findings confirm a subclinical left ventricular dysfunction in both subgroups at the end of our study, which could also be detected by the TDI method at the beginning of the disease progress (Fig. 1).

### LV diastolic function

**Fig. 2:**
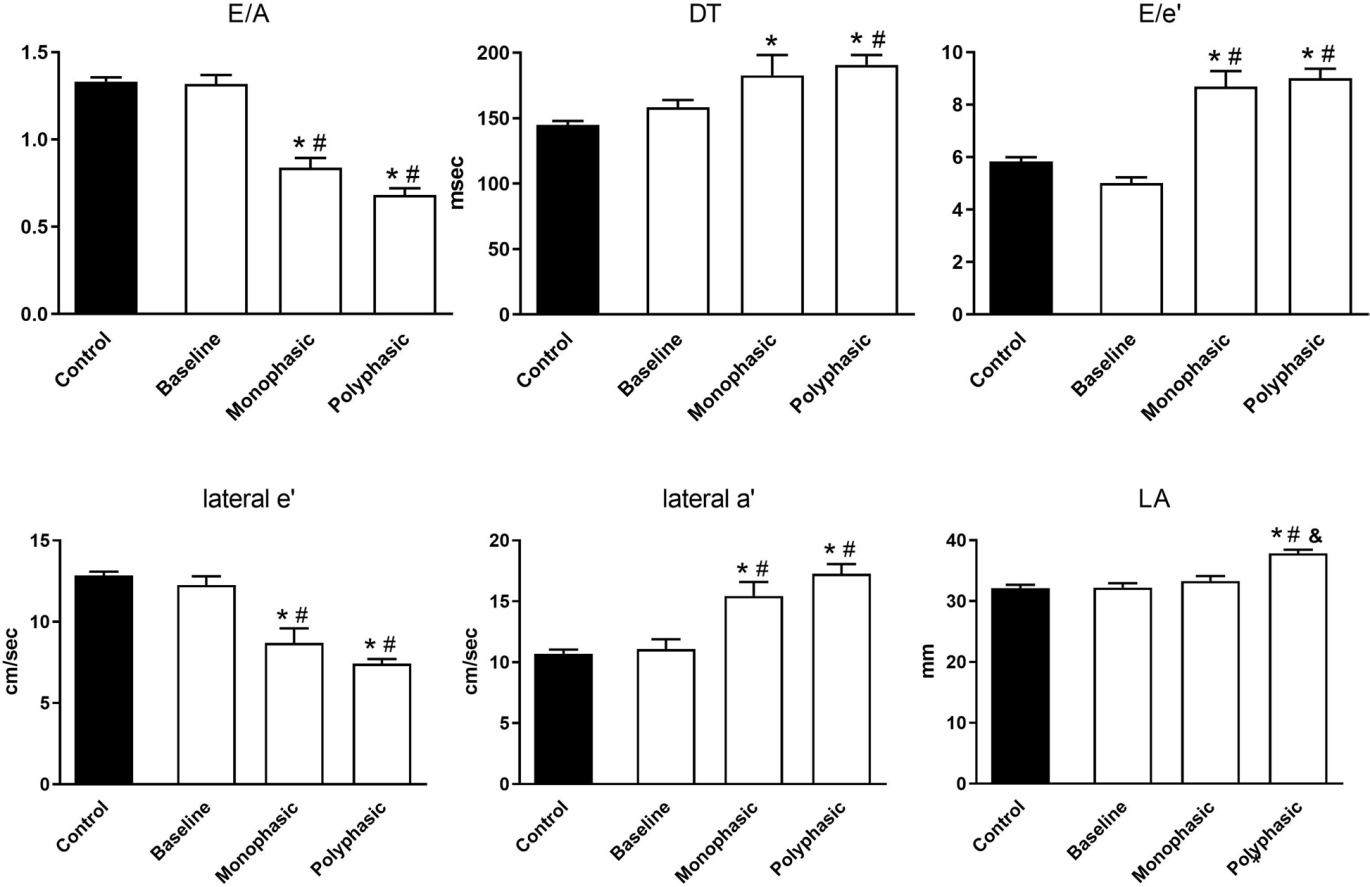
Left ventricular diastolic function during the 2-year follow-up. E: peak early diastolic velocity, A: peak late diastolic velocity, DT: deceleration time of the E wave, lateral e′: early diastolic myocardial relaxation velocity, lateral a′: late diastolic myocardial relaxation velocity. E/A ratio calculated from the transmitral flow was in the normal range (>1) at the time of the diagnosis; however, it decreased significantly with parallel lengthening DT in the 2year follow-up period in the monophasic and the polyphasic group compared to the baseline and control group revealing diastolic dysfunction. Increasing E/e′ ratio during the follow-up period in both groups suggests elevating filling pressure and also supports the development of the diastolic dysfunction. TDI parameters confirm the above observations: lateral e′ decreased, lateral a′ increased significantly in the monophasic and polyphasic groups compared to the baseline and control groups. Additionally, LA diameter was comparable to the control and baseline in the monophasic groups; however, it was found to be significantly larger in the polyphasic group. * *P*<0.05 vs. control, # *P*<0.05 vs. baseline, & *P*<0.05 vs. monophasic

LV diastolic function was evaluated by the characterization of the transmitral inflow Doppler pattern (E/A, DT) and the TDI measurement of the lateral segment of the LV myocardium (lateral e′ and lateral a′ velocities) (Table 2, Fig. 2). Any diastolic abnormalities could not be detected at baseline; however, a grade I diastolic dysfunction appeared both in the monophasic and polyphasic group: significantly lower E/A ratio, a longer DT were measured in the polyphasic and the monophasic group compared to the controls and the baseline timepoint (E/A ratio: 1.33±0.02, 1.32±0.1, 0.84±0.06, 0.68±0.04; DT: 144.7±3.2 msec, 158.3±5.7 msec, 182.8±15.4 msec, 190.8±7.6 msec; control, baseline, monophasic, polyphasic, respectively). Accordingly, the early diastolic lateral myocardial velocity (lateral e′) decreased (12.9±0.2 cm/s, 12.3±0.6 cm/s, 8.7±0.9 cm/s, 7.4±0.3 cm/s; control, baseline, monophasic, polyphasic, respectively) and the late diastolic myocardial velocity (lateral a′) increased significantly in the two disease groups at the end of the follow-up period (10.7±0.3 cm/sec, 11.1±0.8 cm/s, 15.4±1.2 cm/s, 17.3±0.8 cm/s; control, baseline, monophasic, polyphasic, respectively). E/e′ ratio - calculated from the mitral inflow E velocity and the TDI lateral e′ velocity is commonly used to estimate the LV filling pressure. The E/e′ ratio was significantly higher in both groups compared to the controls and the baseline (5.8±0.2, 5.0±0.2, 8.7±0.6, 9.0±0.4; control, baseline, monophasic, polyphasic, respectively). These results show that diastolic dysfunction (grade I – impaired relaxion) appears both in the monophasic and polyphasic groups (Fig. 2). Larger LA diameter supports a further impairment of the diastolic function in the polyphasic group (32.1±0.6 mm, 32.2±0.7 mm, 33.3±0.8 mm, 37.8±0.6 mm; control, baseline, monophasic, polyphasic, respectively).

### RV function and pulmonary artery systolic pressure

**Fig. 3:**
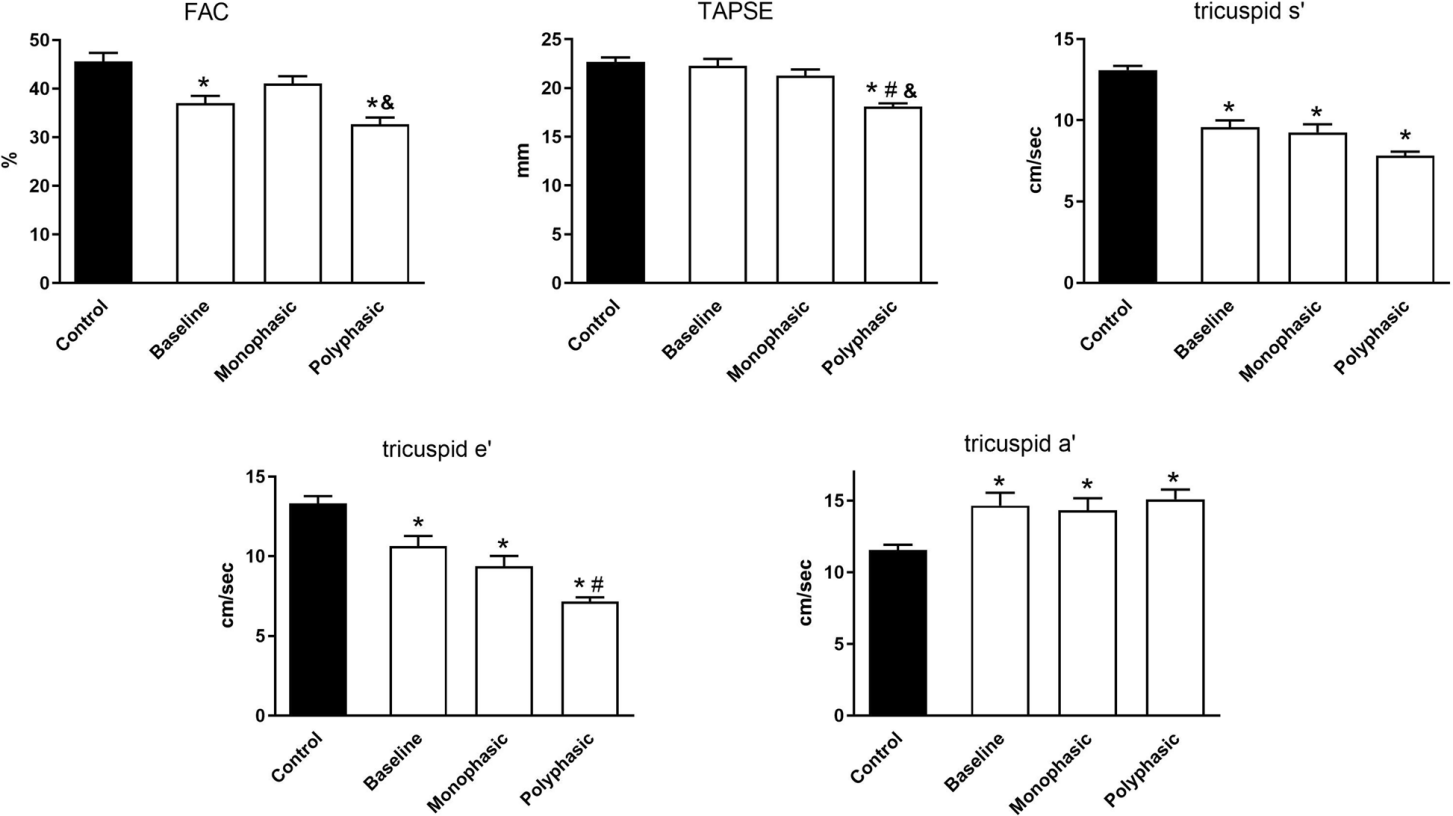
Right ventricular systolic and diastolic function. FAC: fractional area change, TAPSE: tricuspid annulus plane systolic excursion, tricuspid s′: tissue Doppler imaging RV systolic wave velocity, tricuspid e′: early diastolic myocardial relaxation velocity, tricuspid a′: late diastolic myocardial relaxation velocity. Lower FAC and TAPSE reveal RV systolic dysfunction in the polyphasic group; however, tricuspid s′ was lower in the monophasic group as well. Significantly lower TDI tricuspid e′ velocity and higher tricuspid a′ velocity characterized RV diastolic dysfunction. * *P*<0.05 vs. control, # *P*<0.05 vs. baseline, & *P*<0.05 vs. monophasic

FAC, TAPSE and the tricuspid systolic velocity (tricuspid s′) were used to characterize RV function. At baseline attenuated FAC and tricuspid s′ velocities could be measured showing a depressed global RV systolic function (FAC: 45.6±1.8%, 37.0±1.5%, 41.0±1.6%, 32.7±1.4%; tricuspid s′: 13.1±0.3 cm/s, 9.6±0.4 cm/s, 9.3±0.5 cm/s, 7.8±0.2 cm/s; control, baseline, monophasic, polyphasic, respectively); however, the longitudinal RV systolic function was similar to the control group (TAPSE: 22.7±0.5 mm, 22.3±0.7 mm, 21.3±0.7 mm, 18.1±0.3 mm; control, baseline, monophasic, polyphasic, respectively). RV systolic function did not decline further in the monophasic group at the end of the follow-up period; however, an additional deterioration was detected in the polyphasic group as demonstrated by the further decline in each systolic RV parameters. Additionally, an RV diastolic dysfunction was also found in the three disease groups which was the most pronounced in the polyphasic subgroup, characterized by the decrease in the tricuspid early diastolic velocity (tricuspid e′: 13.3±0.5 cm/s, 10.7±0.6 cm/s, 9.4±0.7 cm/s, 7.2±0.3 cm/s; control, baseline, monophasic, polyphasic, respectively) and the increase in the tricuspid late diastolic velocity (tricuspid a': 11.5±0.4 cm/s, 14.6±0.9 cm/s, 14.3±0.9 cm/s, 15.1±0.7 cm/s; control, baseline, monophasic, polyphasic, respectively), similarly as observed in the left heart. These findings present a subclinical RV systolic dysfunction (FAC, TAPSE, tricuspid s′) and a RV diastolic dysfunction (tricuspid e′, a′) which was the most severe in the polyphasic group at the end of the study (Fig. 3). We could not detect any direct, or indirect echocardiographic signs of pulmonary hypertension, in either group (data not shown).

## Discussion

In our recent work, we have conducted a prospective follow-up echocardiographic study which confirmed the changes in the left and right ventricular function in middle-aged PM/DM patients without CV risks factors after 2 years of specific therapy. Echocardiographic alterations were found according to the course of the disease (monophasic vs. polyphasic). In line with other studies [14, 15], a time-related LV diastolic dysfunction (grade I) occurred both in the monophasic and the polyphasic group during the follow-up. However, differences could not be found between the disease subgroups, a tendency towards a more severe diastolic function is suggested by a larger LA and LVED diameter in the polyphasic group. In parallel with the left heart, RV diastolic dysfunction also appeared in the mono- and the polyphasic groups, and it seems to be more severe in the polyphasic group as shown by the significantly lower tricuspid e′ velocity compared to the controls. Additionally, a subclinical left and right ventricular systolic dysfunction could be observed at the end of the study in both subgroups of the PM/DM patients. Moreover, our results suggest more severe systolic cardiac manifestations in the polyphasic group 2 years after the diagnosis.

PM/DM patients are generally classified into four subgroups based on the course of the disease: acute fulminant, monophasic, polyphasic and chronic progressive forms. Monophasic and polyphasic groups were involved in our study since it would be impossible to collect data from patients with acute fulminant disease course and there was not enough time to enrol ones from the chronic progressive group. By definition, no new flare appears in the monophasic group after the first therapy remission while polyphasic patients are characterized by several relapses. More than half of the relapses appear typically in the first 2 years on maintenance therapy. Its severity ranges from subclinical CK elevation to severe clinical relapse. Although the relapse rate does not differ in DM and PM, multiple relapses are more common in DM. The occurrence of the relapses is not related either to the initial severity of the disease or the time between the diagnosis and the beginning of the therapy [19].

Cardiac involvement is a frequent complication in patients with IIM; additionally, it has a prognostic significance. The basic abnormality in the myocardium is inflammation with necrosis and fibrosis irrespective of the disease course, similar to the pathological changes seen in the skeletal muscle. Furthermore, there has been growing evidence suggesting accelerated atherosclerosis in the patients with IIM in the last few years which could be recognized by several noninvasive methods, ie biomarkers [20]. Systematic and local inflammation may either have a direct effect on the myocardium or make the heart more susceptible to traditional risk factors [21]. Vascular alterations affecting the coronary arteries have also been reported such as vasculitis, intimal proliferation, media sclerosis, and microvessel disease with vasospastic angina [22]. Other patophysiological mechanisms—e.g. enhanced chamber stiffness caused by fibrosis or disturbances of the calcium regulation—may cause LV diastolic and systolic failure [23]. Moreover, the specific autoantibodies, anti-Jo, anti-Ro-52 and anti-Ro-60 promote the production of interferon-alpha causing direct myofiber destruction [24]. Interleukin-6, interleukin-1 beta and the tumour necrosis factor (TNF)-alpha may cause myocyte damage via the major histocompatibility complex-1, by local nitric oxide release and myocardial fibrosis [25]. The exact molecular and cellular mechanisms of myocardial dysfunction in PM/DM have not yet been clarified.

**Fig. 4:**
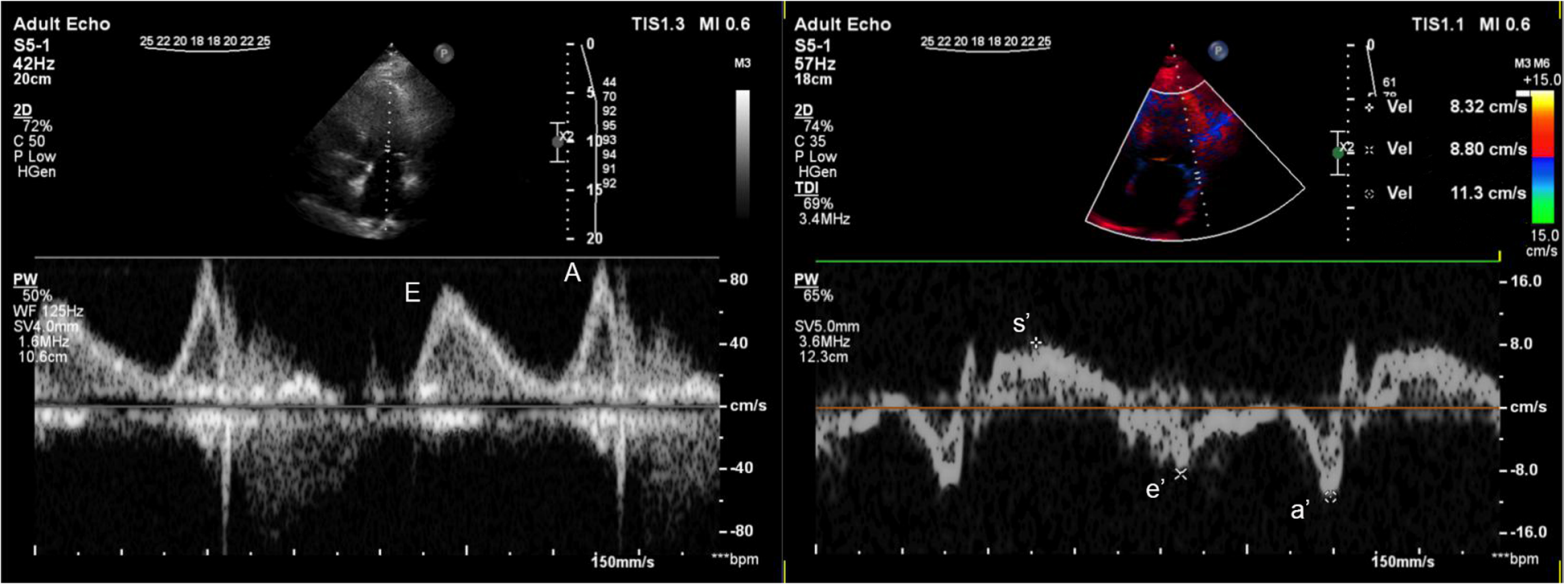
Representative echocardiographic results in polyphasic group after 2 years follow-up. Representative images of echo patterns of mitral inflow from the 4-chamber view in the left panel: impaired relaxation pattern with decreased E wave velocity and increased A wave velocity. Typical tissue Doppler image of the lateral LV wall on the right: decreased lateral s′ (systolic velocity), decreased lateral e′ (early diastolic myocardial relaxation velocity) and increased lateral a′ (late diastolic myocardial relaxation velocity)

Left ventricular diastolic dysfunction is an early feature of cardiac involvement in PM/DM. A mild diastolic dysfunction (grade I - impaired relaxation) with normal filling pressure (A≥E, E/e′≤8, normal LA diameters) appeared in PM/DM patients without CV risk factors after 3-month steroid therapy in our previous study, which suggest intrinsic myocardial manifestation with the fibrotic transformation of the myocardium as the first step of the process [13]. Lu [14] and Wang et al. [15] reported diastolic dysfunction 4.78 and 11.12 months after disease onset in similar middle-aged PM/DM groups. Wang et al. found also LV diastolic dysfunction in DM patients; moreover, they observed an association between transmitral flow alteration and disease duration [15]. A systematic review suggested the role of myocardial fibrosis and recurrent myocarditis in the background [26] since the continuous cardiomyocyte damage, pathologic calcification and the high level of cytokines (e.g. vascular cell adhesion molecule, TNF-alpha) may all contribute to the processes of the cardiac diastolic dysfunction [27]. Furthermore, the development of mild hypertension because of long-term steroid treatment of the patients might also contribute to the pathophysiology. The current work confirmed the presence of subclinical diastolic dysfunction after 2 years follow-up as well. Grade I diastolic dysfunction could be found in both examined patient groups (monophasic and polyphasic); however, a more severe diastolic dysfunction in the polyphasic group (Fig. 4) suggests these patients to be more vulnerable for the clinical manifestation of the diastolic dysfunction.

Recurrence acute myocarditis and myocardial acute phase oedema may cause clinical or subclinical left and right ventricular systolic dysfunction which can result in subepicardial late contrast enhancement on cardiac MRI [27] and can be revealed by TDI and strain rate imaging echocardiographic methods [13, 28]. The traditional systolic parameter, the LVEF is a commonly used conventional parameter to measure changes in the systolic function; however, more sensitive echocardiographic techniques such as TDI and strain rate are more suitable methods for the recognition of the subclinical systolic dysfunction. Guerra et al. first use the two-dimensional speckle-tracking method in the literature of 28 adult patients (mean age: 61.3±13.1 years) with myositis, without symptoms, demonstrating significantly lower RV global systolic strain (RVGLS) and LV global systolic strain (LVGLS) compared with controls [29]. Zhong et al. also have published a three-dimensional speckle-tracking echo study of 60 IIM patients (without clinical manifestation of coronary artery disease, mean age: 51.1±12.6 years) who had significantly diminished LVGLS and RVGLS. In addition, myositis damage index was independently associated with LVGLS and RVGLS [30]. These results correspond with our TDI findings which show subclinical left and right ventricular dysfunction after 2 years of disease onset.

The observed subclinical cardiac involvement seems to be reversible in the PM/DM patients treated with specific medication (high dose steroid and immunosuppression) that we have already suggested in our previous echocardiographic study [13] and also Allanore’s group found reduced myocardial MRI late contrast enhancement as a result of the specific therapy [12, 28]. Beyond echocardiography, CMR would be the best choice for diagnosing cardiac myocarditis and fibrosis. Inflammatory myocarditis is diagnosed by CMR using three tissue characteristics including myocardial oedema, capillary leak and fibrosis [31]. CMR is able to differentiate between myocardial infarction and inflammation since the subendocardial layer is not affected in enhanced delayed sequences in the inflammatory tissue [32]. A number of small series (14–26 patients) have evaluated the role of CMR in detecting myocardial involvement in IIM without cardiac symptoms [6, 33, 34]. Mavrogeni et al. reported epicardial and intramyocardial late gadolinium enhancement (LGE ) typical for past inflammation in 56.3% of the examined 16 PM/DM patients (mean age: 44 years, without clinical CV manifestation, 24 months follow-up) with normal LV volumes and normal LVEF [33]. Khoo et al reported LGE in 9/19 asymptomatic IIM patients: myocardial inflammation, fibrosis or infiltration can be patchy, sub-epicardial and mid-myocardial with CMR [35]. No studies have compared echocardiography with CMR in IIM. Further long-term studies involving a larger number of patients and multimodality imaging would be necessary for definitive conclusions.

The possible limitations of the study should be acknowledged: relative small case number without cardiac troponin-I levels, CMR and myocardial biopsy results. TDI measurement uses the Doppler method so data are strongly affected by the angle of the ultrasound beam. To avoid errors arising from this fact, all parameters were measured in three independent heart cycles and the mean data were calculated.

## Conclusion

Our results demonstrate that the early echocardiographic changes detected in myositis patients at the diagnosis show progression in the polyphasic group after 2 years with a more severe left and right ventricular diastolic dysfunction and newly appearing subclinical systolic dysfunction. This is possibly due to the recurrent inflammations, myocarditis and myocardial fibrosis. Echocardiographic TDI is a suitable tool (beyond the speckle-tracking strain rate method and CMR) for the diagnosis and the follow-up of the early cardiac abnormalities in PM/DM patients. This method can be useful to differentiate cardiac variables between monophasic and polyphasic groups and to plan for therapeutic driving.

## Data Availability

The datasets generated during and/or analysed during the current study are available from the corresponding author on reasonable request.
